# Protein carbonyl concentration as a biomarker for development and mortality in sepsis-induced acute kidney injury

**DOI:** 10.1042/BSR20171238

**Published:** 2018-01-25

**Authors:** Nara Aline Costa, Ana Lúcia Gut, Paula Schmidt Azevedo, Suzana Erico Tanni, Natália Baraldi Cunha, Ana Angelica Henrique Fernandes, Bertha Furlan Polegato, Leonardo Antonio Mamede Zornoff, Sergio Alberto Rupp de Paiva, André Luís Balbi, Daniela Ponce, Marcos Ferreira Minicucci

**Affiliations:** 1 Department of Internal Medicine, Botucatu Medical School, UNESP – Univ Estadual Paulista, Botucatu, Brazil; 2 Department of Chemistry and Biochemistry, Institute of Biological Sciences, UNESP – Univ Estadual Paulista, Botucatu, Brazil

**Keywords:** acute kidney injury, oxidative stress, protein carbonyl, sepsis

## Abstract

The objective of the present study was to evaluate protein carbonyl concentration as a predictor of AKI development in patients with septic shock and of renal replacement therapy (RRT) and mortality in patients with SAKI. This was a prospective observational study of 175 consecutive patients over the age of 18 years with septic shock upon Intensive Care Unit (ICU) admission. After exclusion of 46 patients (27 due to AKI at ICU admission), a total of 129 patients were enrolled in the study. Demographic information and blood samples were taken within the first 24 h of the patient’s admission to determine serum protein carbonyl concentrations. Among the patients who developed SAKI, the development of AKI was evaluated, along with mortality and need for RRT. The mean age of the patients was 63.3 ± 15.7 years, 47% were male and 51.2% developed SAKI during ICU stay. In addition, protein carbonyl concentration was shown to be associated with SAKI. Among 66 patients with SAKI, 77% died during the ICU stay. Protein carbonyl concentration was not associated with RRT in patients with SAKI. However, the ROC curve analysis revealed that higher levels of protein carbonyl were associated with mortality in these patients. In logistic regression models, protein carbonyl level was associated with SAKI development (OR: 1.416; 95% CI: 1.247–1.609; *P*<0.001) and mortality when adjusted by age, gender, and APACHE II score (OR: 1.357; 95% CI: 1.147–1.605; *P*<0.001). In conclusion, protein carbonyl concentration is predictive of AKI development and mortality in patients with SAKI, with excellent reliability.

## Introduction

Sepsis is defined as life-threatening organ dysfunction caused by a dysregulated host response to infection. According to the Sepsis-3 definition, septic shock is a subset of sepsis with circulatory and metabolic dysfunction associated with a higher risk of mortality [1]. Despite increased understanding of the pathogenesis of sepsis and the creation of “bundles” of care, the septic shock mortality rate remains high, killing as many as one in four patients [2]. Sepsis-induced acute kidney injury (SAKI) is one of the most serious and frequent complications of sepsis, occurring in approximately 51–64% of patients with sepsis [3]. The development of SAKI increases the risk of in-hospital death 6- to 8-fold, and, among survivors, the risk of progression to chronic kidney disease (CKD).

However, until now, no therapeutic measures have been available to prevent or treat SAKI. The absence of an early biomarker and delays in treatment initiation could explain the lack of therapeutic interventions in SAKI [4]. In the past few years, several biomarkers have been studied; however, only a few of them provide insights into the pathophysiology of the disease and none of them leads to an important change in therapeutic approach [5].

To date, microcirculatory dysfunction, inflammation, and adaptive responses of tubular epithelial cells to injury, such as oxidative stress, have been considered the hallmarks of SAKI [3,4,6]. Thus, we deemed the oxidative stress markers to be an interesting research target in this scenario. Several biomarker had been studied in critically ill patients such as erythrocyte superoxide dismutase (SOD) 1 activity, advanced oxidation protein products, and monocyte HLA_DR expression [7–10]. In a previous study by our group, we showed that erythrocyte SOD 1 activity was associated with AKI development in patients with septic shock [7]. SOD is considered the first line of defense against reactive oxygen species (ROS); however, it is only one of the antioxidant system enzymes and does not reflect oxidative tissue injury.

Oxidative tissue damage can be measured by different products derived from proteins, lipids, and DNA. Protein carbonyl groups are markers of protein oxidative damage that are formed early during the sepsis process and are more stable than the lipid peroxidation products [11,12]. Experimental and clinical studies showed higher levels of protein carbonyl concentration in acute kidney injury (AKI) [13–16]. In addition, we recently showed, using the same dataset that we used in the present study, that protein carbonyl, but not serum malondialdehyde, concentration is associated with ICU mortality in patients with septic shock [17]. However, protein carbonyl concentration has not yet been evaluated as a biomarker of SAKI development or SAKI mortality.

Thus, the objective of the present study was to evaluate the protein carbonyl concentration as a predictor of AKI development in patients with septic shock, and of renal replacement therapy (RRT) and mortality in patients with SAKI.

## Methods

The present study was a subanalysis of previous studies that analyzed oxidative stress markers as early markers of septic shock mortality and septic AKI development [7,17]. This prospective observational study was conducted from May, 2014 to June, 2015 and involved patients admitted to the Intensive Care Unit (ICU) of our institution. The present study was conducted according to the guidelines established by the Declaration of Helsinki, and all procedures involving human patients were approved by the Ethics Committee of our institution (30457414.7.0000.5411). Written informed consent was obtained from all patients.

Patients were eligible for enrollment if they were 18 years or older and had septic shock on ICU admission. Exclusion criteria included patients with AKI at ICU admission, patients with stage 4 or 5 chronic kidney disease (CKD) (creatinine clearance lower than 30 ml/min/1.73 m^2^), a delay in septic shock diagnosis (longer than 24 h), pregnant women, patients with confirmed brain death, patients in palliative care, and those who used vasoactive drugs for less than 24 h.

Upon admission, patient demographic information, along with the Acute Physiology and Chronic Health Evaluation (APACHE II) and the Sequential Organ Failure Assessment (SOFA) scores were recorded. Blood samples were taken within the first 24 h of admission to determine serum protein carbonyl concentrations. Patients were followed during their ICU stay and the development of AKI was evaluated. Among the patients who developed AKI, mortality and the need for renal replacement therapy (RRT) were also evaluated.

Septic shock was defined according to the Survival Sepsis guidelines [18], and AKI was defined according to Kidney Disease Improving Global Outcomes (KDIGO) criteria, using the increase in serum creatinine ≥0.3 mg/dl within 48 h or increase in serum creatinine ≥1.5 times from baseline within 7 days [19]. The baseline creatinine was defined as the lowest creatinine value in the last 6 months before AKI or, for those without this measurement, the lowest value achieved during hospitalization in the absence of dialysis [20,21]. CKD was defined as a glomerular filtration rate lower than 60 ml/min/1.73 m^2^ using baseline creatinine and the CKD Epidemiology Collaboration equation (CKD-EPI) [22].

### Serum protein carbonyl concentration

Protein carbonyl was analyzed through the reaction with dinitrophenylhydrazine and the formation of a Schiff base, according to the method described by Reznick and Packer [23]. Serum samples were incubated in presence of 10 mmol/l dinitrophenylhydrazine in the dark at room temperature for 1 h, vortexing every 10 min. Proteins were precipitated with ice-cold 50% trichloroacetic acid (wt./vol.) and centrifuged (10000 ***g*** for 10 min). The pellets were washed three times with ethanol-ethyl acetate (1:1; vol./vol.) mixture and resuspended in 6 mol/l guanidine hydrochloride at 37°C for 10 min. The level of protein carbonyl was quantified spectrophotometrically at 360 nm using an extinction coefficient 22,000 M^−1^ cm^−1^

### Laboratorial analysis

Hemograms were performed with a Coulter STKS hematologic autoanalyzer (Luton/Bedfordshire, U.K.). Total serum levels of C-reactive protein (CRP), albumin, glycemia, creatinine, and urea were measured using the dry chemistry method (Ortho-Clinical Diagnostics VITROS 950®, Johnson & Johnson), and lactate was measured using a Roche OMNI® S Blood Gas Analyzer.

### Statistical analysis

Data are expressed as the mean ± SD or the median (including the lower and upper quartiles). Statistical comparisons between groups for continuous variables were performed using the Student’s *t*-test for parameters with a normal distribution. If data were not normally distributed, comparisons between groups were made using the Mann–Whitney *U*-test. Fisher’s test or the Chi-square test was used for all categorical data. Spearman correlation was performed to analyze the association between continuous variables. Receiver operating characteristic (ROC) curve analysis was performed to determine the performance of protein carbonyl concentration in the prediction of AKI development and SAKI mortality. A logistic regression model was used to predict SAKI development and mortality. Protein carbonyl concentration was tested as a continuous variable, and it was adjusted by age, gender, APACHE II score, and CKD. Data analysis was performed using SigmaStat software for Windows v3.5 (Systat Software Inc., San Jose, CA, U.S.A.). *P*-values less than 0.05 were considered statistically significant.

## Results

**Figure 1:**
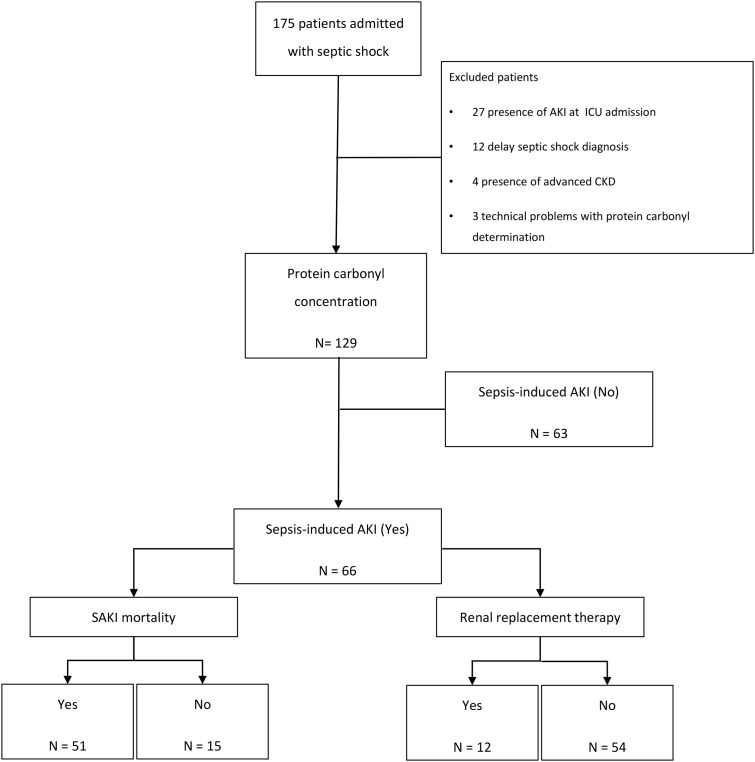
Flow diagram of studied patients

**Figure 2:**
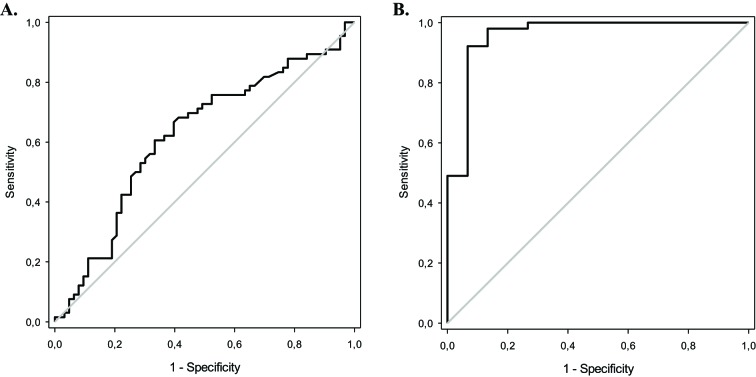
Receiver operating curves analysis (**A**) ROC curve for the association between protein carbonyl concentrations and sepsis-AKI development in 129 patients with septic shock (AUC: 0.621; 95% CI: 0.523–0.719; *P*=0.018). (**B**) ROC curve for the association between protein carbonyl concentrations and sepsis-AKI mortality in 66 patients (AUC: 0.958; 95% CI: 0.892–1.000; *P*<0.001).

During the study, 175 consecutive patients with a diagnosis of septic shock in the ICU were admitted; however, 46 patients were excluded (presence of AKI at ICU admission: 27 patients; delay in septic shock diagnosis: 12 patients; presence of advanced chronic kidney disease: 4 patients; technical problems with determining protein carbonyl concentration: 3 patients). Thus, a total of 129 patients were ultimately evaluated (Figure 1). The mean patient age was 63.3 ± 15.7 years, 47% were male and the median protein carbonyl concentrations were 24.5 (13.3–32.7) nmol/ml. Among these patients, 51.2% developed SAKI during the ICU stay. The patients who developed SAKI presented with higher APACHE II and SOFA scores, CRP, urea, and creatinine values, had more CKD and increased mortality rate. In addition, 18.2% of the patients with SAKI needed RRT, and they presented with lower levels of hemoglobin. It is also interesting to observe that protein carbonyl concentration had a positive correlation with SOFA score (*r* = 0.235; *P*=0.007). Moreover, protein carbonyl concentrations were higher in patients who developed SAKI (Table 1). The AUC of protein carbonyl concentration was 0.621, with a 95% CI of 0.523–0.719 (Figure 2).

**Table 1:** Demographic, clinical, and laboratory data of 129 patients with septic shock

Variables	Sepsis-induced AKI development		*P*-value
	Yes (*n*=66)	No (*n*=63)	
Age (years)	67.0 (58.3–75.3)	64.0 (51.0–73.0)	0.22
Male, *n*° (%)	32 (48.5)	29 (46.0)	0.92
APACHE II score	18.8 ± 6.0	16.1 ± 6.5	0.01
SOFA score	10.0 (8.0–12.0)	8.0 (7.0–10.0)	<0.001
RBC transfusions, *n*° (%)	35 (53.0)	28 (44.4)	0.42
Sepsis focus, *n*° (%)			0.45
Respiratory	41 (62.1)	32 (50.8)	
Abdominal	16 (24.2)	16 (25.4)	
Urinary	3 (4.6)	4 (6.3)	
Others	6 (9.1)	11 (17.5)	
RRT, *n*° (%)	12 (18.2)	0 (0)	0.001
CKD, *n*° (%)	57 (86.4)	7 (11.1)	<0.001
ICU mortality, *n*° (%)	51 (77.3)	35 (55.6)	0.015
Lactate (mmol/l)	2.2 (1.4–3.6)	2.1 (1.2–3.5)	0.79
Hemoglobin (g/dl)	11.0 (9.1–12.0)	11.6 (10.0–12.7)	0.04
Hematocrit (%)	32.1 ± 6.5	34.0 ± 5.6	0.08
Leukocytes (10^3^/mm^3^)	16.7 (12.2–21.8)	16.3 (12.2–23.6)	0.86
Glycemia (mg/dl)	145 (118–186)	145 (115–190)	0.90
CRP (mg/dl)	35.9 (27.7–44.2)	31.5 (8.4–39.6)	0.04
Protein carbonyl (nmol/ml)	27.8 (15.9–33.1)	19.4 (12.2–29.1)	0.018
Albumin (g/dl)	2.2 (2.0–2.5)	2.1 (1.8–2.8)	0.56
Urea (mg/dl)	97 (68–159)	53 (32–88)	<0.001
Creatinine (mg/dl)	2.1 (1.6–2.7)	0.7 (0.5–1.1)	<0.001

Abbreviations: APACHE II, Acute Physiology and Chronic Health Evaluation; CKD, chronic kidney disease; CRP, C-reactive protein; ICU, Intensive Care Unit; MV, mechanical ventilation; RBC, red blood cells; RRT, renal replacement therapy; SOFA, Sequential Organ Failure Assessment. Data are expressed as a mean ± SD, median (including the lower and upper quartiles), or percentage.

The demographic and clinical data of the 66 patients with SAKI are presented in Table 2. Among these patients, 77% died during ICU stay, 48% were male, and the median protein carbonyl concentrations were 27.8 (15.9–33.1) nmol/ml. Only the APACHE II and SOFA scores were higher in patients who died during ICU stay. Upon evaluation of the laboratory data, only higher levels of protein carbonyl were associated with mortality in SAKI (Table 2). On the other hand, protein carbonyl concentration was not associated with RRT in patients with SAKI [RRT (*n*=12): 30.8 (24.1–35.1) nmol/ml; non-RRT (*n*=54): 27.1 (13.6–33.1) nmol/ml; *P*=0.25].

**Table 2:** Demographic, clinical, and laboratory data of 66 patients with septic shock who developed acute kidney injury during ICU stay

Variables	ICU mortality		*P*-value
	Yes (*n*=51)	No (*n*=15)	
Age (years)	67.0 (60.0–76.0)	64.0 (51.0–75.0)	0.40
Male, *n*° (%)	27 (52.9)	5 (33.3)	0.30
APACHE II score	20.0 ± 5.9	14.8 ± 4.7	0.003
SOFA score	10.7 ± 2.6	8.6 ± 1.7	0.005
RBC transfusions, *n*° (%)	28 (54.9)	7 (46.7)	0.79
Sepsis focus, *n*° (%)			0.46
Respiratory	32 (62.7)	9 (60.0)	
Abdominal	11 (21.6)	5 (33.3)	
Urinary	2 (3.9)	1 (6.7)	
Others	6 (11.8)	0 (0)	
RRT, *n*° (%)	11 (21.6)	1 (6.7)	0.27
CKD, *n*° (%)	43 (84.3)	14 (93.3)	0.67
Lactate, (mmol/l)	2.2 (1.3–3.4)	2.4 (1.5–4.5)	0.83
Hemoglobin, (g/dl)	10.9 (9.1–11.8)	11.2 (9.0–12.3)	0.65
Hematocrit (%)	32.1 ± 6.4	32.1 ± 7.0	0.99
Leukocytes (10^3^/mm^3^)	16.6 (12.1–23.9)	17.0 (12.2–19.4)	0.62
Glycemia (mg/dl)	138 (116–182)	153 (132–186)	0.20
CRP (mg/dl)	36.2 (26.7–44.1)	35.5 (30.2–44.3)	0.59
Protein carbonyl (nmol/ml)	29.5 ± 6.80	11.3 ± 6.32	< 0.001
Albumin (g/dl)	2.2 (1.9–2.5)	2.3 (2.0–2.7)	0.40
Urea (mg/dl)	102 (70–160)	69 (57–130)	0.18
Creatinine (mg/dl)	2.2 ± 0.9	2.1 ± 0.8	0.81

Abbreviations: APACHE II, Acute Physiology and Chronic Health Evaluation; CKD, chronic kidney disease; CRP, C-reactive protein; ICU, Intensive Care Unit; MV, mechanical ventilation; RBC, red blood cells; RRT, renal replacement therapy; SOFA, Sequential Organ Failure Assessment. Data are expressed as a mean ± SD, median (including the lower and upper quartiles), or percentage.

The ROC curve analysis revealed that higher levels of protein carbonyl were associated with mortality in these patients, with an excellent AUC. (AUC: 0.958; 95% CI: 0.892–1.025; *P*<0.001) at the cutoff of >25.1 nmol/ml [sensibility: 78.4% (95% CI: 67.1–89.7%); specificity: 93.3% (95% CI: 80.7–105.9%); positive predictive value: 97.6% (95% CI: 92.9–102.3%); negative predictive value: 56.0% (95% CI: 37.0–75.0%)] (Table 3) (Figure 2).

**Table 3:** Cross tabulation of protein carbonyl concentration and ICU mortality

Protein carbonyl concentration	ICU mortality		Total
	Yes	No	
>25.1 nmol/ml	40	1	41
≤25.1 nmol/ml	11	14	25
Total	52	15	66

In the logistic regression models, protein carbonyl level was associated with SAKI development (OR: 1.416; 95% CI: 1.247–1.609; *P*<0.001) (Table 4), and mortality when adjusted by age, gender, APACHE II score, and CKD (OR: 1.357; 95% CI: 1.147–1.605; *P*<0.001) (Table 5).

**Table 4:** Logistic regression model for the prediction of SAKI development during ICU stay in 129 patients with septic shock

Variable	OR	CI 5–95%	*P*-value
Protein carbonyl (nmol/ml)*	1.044	1.008–1.081	0.015
Protein carbonyl (nmol/ml)**	1.416	1.247–1.609	<0.001

* Unadjusted; **Adjusted by gender, age, APACHE II score, and chronic kidney disease.

**Table 5:** Logistic regression model for the prediction of mortality in SAKI during ICU stay in 66 patients

Variable	OR	CI 5–95%	*P*-value
Protein carbonyl (nmol/ml)*	1.357	1.163–1.583	<0.001
Protein carbonyl (nmol/ml)**	1.357	1.147–1.605	<0.001

* Unadjusted; **Adjusted by gender, age, APACHE II score, and chronic kidney disease.

## Discussion

The aim of the present study was to evaluate protein carbonyl concentration as a predictor of AKI development in patients with septic shock and as a predictor of RRT and mortality in patients with SAKI. According to our results, protein carbonyl concentration is predictive of AKI development and mortality in patients with SAKI. Notably, protein carbonyl concentration showed excellent reliability in the prediction of SAKI mortality (AUC: 0.958).

Until now, prognostic biomarkers have mainly been used to stratify the observation level of patients. In order to provide information regarding immediate organ prognosis and to influence the therapeutic strategy, these biomarkers must add to the understanding of the pathophysiology of the disease [5]. Despite microcirculatory dysfunction and inflammation, SAKI is also associated with an adaptive response to injury [3,4,6]. Gomez et al. [6] hypothesized that oxidative stress is the trigger for the adaptive response of the tubular epithelial cells, which is characterized by reprioritizing energy expenditure, down-regulating metabolism, and undergoing cell cycle arrest. Therefore, although oxidative stress is a generalized phenomenon in sepsis, it plays an important role in SAKI pathology. Likewise, it could be an interesting target for interventions.

In our study’s prognostic scores, inflammation, the presence of CKD, and hemoglobin levels were all associated with SAKI development. However, interestingly, the markers of tissue perfusion (lactate) and inflammation (CRP), which supposedly participate in SAKI pathophysiology, were not in fact associated with SAKI mortality. Moreover, protein carbonyl concentration and APACHE II and SOFA scores were the only laboratory and demographic data associated with mortality in patients with SAKI.

The formation of protein carbonyl groups has been previously studied in experimental and clinical studies of AKI. Himmelfarb et al. [14] showed that protein carbonyl content was higher in critically ill patients with AKI compared with healthy subjects, patients with end-stage renal disease, and critically ill patients without AKI. Our group also showed that serum protein carbonyl concentrations were higher in patients with septic shock who died during ICU stay [24]. In addition, we also showed that protein carbonyl, but not malondialdehyde, concentration is associated with ICU mortality in patients with septic shock [17]. However, serum protein carbonyl levels have not yet been studied as an early marker of SAKI development and mortality.

In the present study, we also showed that protein carbonyl concentration is associated with SAKI development. However, the most interesting finding is that protein carbonyl levels were also associated with mortality in patients who developed SAKI, even when adjusted by age, gender, and APACHE II score in multivariate analysis, with an excellent performance. In addition, the positive predictive value was 97.6%, suggesting that patients with SAKI and protein carbonyl concentration values higher than 25.1 nmol/ml had a high probability of death.

Protein carbonyl groups are early markers of protein oxidative damage. Indeed, Andresen et al. [12] previously demonstrated, in patients with septic shock, that malondialdehyde, a lipoperoxidation marker, increases over time, whereas protein oxidative damage reaches its peak early upon ICU admission. The variable kinetics of macromolecule oxidation products could be explained by the different timings of their formation and detoxification and by the nature of the reactive oxygen species that are produced [11,12]. Thus, among other biomarkers of oxidative damage, we believe that protein carbonyl concentration has a particularly interesting profile. In addition, it is a simple, fast, and inexpensive assessment method that could be quickly incorporated into routine clinical practice.

Although protein carbonyl concentration was shown to be a useful tool to predict SAKI mortality, our study has limitations. First, our study included a small sample size and patients from a unique medical center. Second, AKI was defined based on serum creatinine levels and not on urine output. Third, although the cutoff of protein carbonyl associated with mortality had excellent specificity, it had low negative predictive value. In addition, we did not evaluate procalcitonin levels, and we did not know the time of disease initiation. Nevertheless, we believe that our study imparts important information regarding SAKI biomarkers. Further studies are necessary to validate the predictive performance of protein carbonyl concentration.

## Conclusions

In conclusion, protein carbonyl concentration predicts AKI development and mortality in patients with SAKI. It is important to highlight that protein carbonyl concentration had an excellent performance in the prediction of SAKI mortality.

## Abbreviations

95% CI: 95% confidence interval
AKI: acute kidney injury
APACHE II score: Acute Physiology and Chronic Health Evaluation II score
CKD: chronic kidney disease
CRP: C-reactive protein
GFR: glomerular filtration rate
ICU: Intensive Care Unit
KDIGO: Kidney Disease Improving Global Outcomes
OR: odds ratio
ROS: reactive oxygen species
RRT: renal replacement therapy
SAKI: sepsis-induced acute kidney injury
SOFA score: Sequential Organ Failure Assessment score
SOD 1: superoxide dismutase 1
